# Comparative Plasma Exposure of Albendazole after Administration of Rapidly Disintegrating Tablets in Dogs

**DOI:** 10.1155/2013/920305

**Published:** 2013-08-25

**Authors:** Silvina G. Castro, Alicia Dib, Gonzalo Suarez, Daniel Allemandi, Carlos Lanusse, Sergio Sanchez Bruni, Santiago D. Palma

**Affiliations:** ^1^Department of Pharmacy, Faculty of Chemical Sciences, UNITEFA, Universidad Nacional de Córdoba, 5000 Córdoba, Argentina; ^2^Consejo Nacional de Investigaciones Científicas y Técnicas-CONICET, Argentina; ^3^Laboratory of Pharmacology, Faculty of Veterinary Medicine, Universidad de la Republica, 11600 Montevideo, Uruguay; ^4^Laboratory of Pharmacology, Faculty of Veterinary Medicine, CIVETAN-UNCPBA, 7000 Tandil, Argentina

## Abstract

The main objectives of this study were (a) to evaluate the *in vitro* performance of the rapid disintegration tablets as a way to improve the solid dispersions and (b) to study the *in vivo* pharmacokinetics of the albendazole modified formulation in dogs. Rapid disintegration of tablets seems to be a key factor for efficiency of solid dispersions with regard to improvement of the albendazole bioavailability. The *in vivo* assays performed on dogs showed a marked increase in drug plasma exposure when albendazole was given in solid dispersions incorporated into rapid disintegration tablets compared with conventional solid dosage form.

## 1. Introduction

Benzimidazoles (BZD) form a family of related anthelmintic compounds and their metabolites/derivatives, which are widely used in antiparasite therapy in both veterinary and human medicine. 

The parent drugs, febendazole (FBZ) and albendazole (ABZ), contain a sulphur atom as a sulphide at position 5 of the BZD molecule. These sulphides are subjected, mainly in the liver, to phase I reactions (oxidation), catalysed by the flavin monooxygenase (FMO) and the cytochrome P-450 (Cyt P-450) enzyme systems to form sulfoxide (SO) metabolites, with FBZSO (oxfendazole; OFZ) and ABZSO being the primary, pharmacologically active metabolites generated [1, 2]. In a second metabolic reaction (sulphonation), catalysed by the Cyt P-450 system OFZ and ABZSO are transformed into inactive sulphone (SO_2_) metabolites, namely, FBZSO_2_ and ABZSO_2_, respectively [2]. 

Several studies have suggested that only limited rates of dissolution and absorption of BZD anthelmintics are achieved in cat, dog, and human. Consequently, these compounds may need to be given at higher doses or as multiple administrations in order to provide adequate therapeutic concentrations to give an acceptable anthelmintic efficacy [3–5]. Therefore, new pharmaceutical alternatives are necessary for increasing the BZD drug exposure to shorten the multiple administrations. According to the biopharmaceutical classification system (BCS), ABZ is a BCS class II compound [6] with an aqueous solubility of less than 5 *μ*g/mL and many investigations have suggested that dissolution of ABZ is the rate-limiting step for its absorption [7]. It is thus desirable to enhance the dissolution rate of the drug in order to increase its rate of absorption. 

The rate at which a solid dissolves is directly proportional to the surface area of drug exposed to the dissolution medium and is given by the Noyes-Whitney equation [8].

Solid dispersions (SDs), which are defined as molecular mixtures of poor water-soluble drugs and hydrophilic carriers, have been proposed as alternatives for improving the dissolution rate of these kinds of drugs [9], with ABZ dissolution having previously been assayed at our laboratory using solid dispersions (SDs) with poloxamer 188 (P 188) as carrier. Although the proportion of P 188 incorporated in SDs had a marked influence on ABZ release during the initial stage (5 min) of the dissolution process. This behavior was not observed for physical mixtures (PMs) of both components. In this context, the material obtained was characterized for the dissolution rate of ABZ, and some mechanisms by which P 188 may act as an efficient carrier in SDs [10] were also explored. However, after an *in vivo* assessment in dogs, the drug plasma exposure of a suspension containing ABZ or ABZ formulated in a simple solid dispersion (capsules) was not significantly augmented [11]. The rise in the *in vitro* dissolution rate may have been attributed to an increase in the dissolution surface area, as well as an improvement in wettability and ABZ solubilization as a consequence of carrier dissolution. 

Any favorable effects of P 188 are hidden by the gelation properties of this material as the temperature is raised [12]. In this way, when solid dispersions are placed in water at 37°C, gel formation is expected [13–15] which may affect the kinetic release mainly due to the increase in viscosity. This fact becomes particularly evident when SDs are vehiculised into capsule, since compaction is produced as a consequence of the filling process [9]. It was clear that compaction played a central role in the potential effectiveness of DSs. 

Furthermore, in previous work we have carried out a pharmacokinetic study in mice using multiparticulate (uncompacted) DSs and P188 as carrier. An increased systemic availability (*P* < 0.001) was obtained when ABZ was administered as ABZ-P 188 SDs, with a 50% enhancement in systemic exposure (AUC values) compared to treatment with an ABZ suspension. Consistently, the Cmax increased 130% (*P* < 0.001) following treatment with P 188 based SD ABZ formulation [16]. In addition, the promising results concerning the potential effectiveness of SDs based on P 188 for improvement of ABZ Bd and the utility of pharmacokinetic studies based on mice model for preliminary screen studies are worthy instead of using superior species (dogs).

Based on these observations, we hypothesize that the design of a solid dosage form with a much faster disintegration would prevent or at least minimize any deleterious gelation effects of P 188 on the ABZ dissolution rate.

Currently, the design and development of rapidly disintegrating tablets (RDTs) is acquiring relevance due to the advantages of this system, such as easier swallowing and bioavailability improvement. The design of RDTs is based on the selection of adequate excipients which allow fast tablet disintegration (few seconds), even with very low amounts of water. The key properties of these tablets are the fast absorption or wetting of water into the tablets and the disintegration of associated particles into individual components for fast dissolution. This requires that excipients should have a high wettability and the tablet structure should also have a highly porous network. In order to achieve these objectives, conventional technologies have been addressed such as tablet molding, freeze drying, direct compression, granulation, and spray drying [17]. Likewise, innovative patented technologies have also been developed. Examples of these are Zydis, Orasolv, Durasolv, Wowtab, Ceform, Flashtab, Pharmaburst, Shearform, Ziplet, Oraquick, and Frosta [18]. 

In the present study we designed a formulation to use a marketed coprocessed excipient that had appropriate properties. In our case, we utilized Ludiflash as the main component of the formulae. This material is able to produce a very fast rupture of the tablet integrity after wetting, with consequent disintegration of the tablet into fine particles. In this context, the main objectives of this study were (a) to evaluate of the performance of the RDTs as a way to improve the SDs *in vitro* and (b) to study the *in vivo* pharmacokinetics of this ABZ modified formulation in dogs.

## 2. Materials and Methods

### 2.1. *In Vitro* Assessment: Materials

The following materials were used for the preparation of the solid dispersions: ABZ (Pharmaceutical grade, Parafarm, Buenos Aires, Argentina) POLOXAMER 188 (BASF, Germany). All other reagents were of analytical grade.

For the preparation of tablets, the following excipients were used: lactose, microcrystalline cellulose, sodium crosscarmellose, colloidal silicon dioxide, magnesium stearate (Parafarm, Buenos Aires, Argentina), and Ludiflash (BASF, Ludwigshafen, Germany).

### 2.2. Methods

#### 2.2.1. Preparation of Solid Dispersions

SDs were prepared by melting the carrier (P 188) at 63°C and with posterior dispersion of ABZ at 50% w/w. The mixtures were homogenized by stirring during heating and dispersion. Then, the semisolid dispersion was rapidly cooled and pulverized. The 212-micron particle size fraction was obtained by sieving and was maintained (8°C) in a screw-capped glass vial until use. 

#### 2.2.2. Development of Solid Dosage Form

The blend of powders was compressed for 5 s in a hydraulic press (Delfabro, Argentina) at 1000, 1500, and 2000 mPa for conventional tablets (CTs) and at 250, 500, and 1000 mPa for RDTs with 13.0 mm flat punches. Tablet hardness was measured on recently prepared tablets using an electronic hardness tester (AVIC, Argentina). Two tablets formulations (Table 1) containing 200 mg of the drug were prepared by direct compression, with the tableting process, as well as subsequent storage of the RDT and CTs, taking place in a conditioned room at 21°C and 45% RH.

#### 2.2.3. Physical Mechanical Properties

*Density and Compressibility.* To determine the density of the powdered blends, the material was gently poured into a 10 cm^3^ graduate cylinder, with bulk density (BD) being calculated as the ratio between the weight (g) and volume (cm^3^). To determine the ultimate tap density (TD), the cylinder was tapped over a 1.0-inch vertical drop, at 1 s interval, until no measurable change in volume was noticed. The compressibility of the powder was evaluated using the Hausner ratio (HR) and Carr's index (CI) [19] according to the following equations:

(1) $$\begin{array}{c} \text{HR}=\frac{\text{TD}}{\text{BD}},\\ \text{CI}=\frac{\text{TD}-\text{BD}}{\text{TD}}\times 100. \end{array}$$

*Angle of Repose (α* *).* The dynamic *α* for each mixture of powders was determined by the funnel method as described in the literature [20].

#### 2.2.4. Dissolution Test

Dissolution tests of RDTs and CTs were performed using USPXXIV dissolution apparatus 2 (SOTAX AT 7 smart). The rotational paddle speed was set at 50 rpm, and the temperature remained constant at 37 ± 0.5°C. The assayed amount of ABZ was 200 mg per tablet for all experiments.

As dissolution medium of 900 mL 0.1 N HCl solution was used, five-milliliter aliquots were withdrawn at predetermined time intervals during 1 h, and the same amount of fresh medium was added in order to keep the volume constant throughout the test. The samples were filtered, and the concentration of dissolved drug was measured at 297 nm using a UV-vis spectrophotometer (Termo Evolution 300).

All measurements were performed in triplicate. In a previous test, we verified that the presence of carriers dissolved in the dissolution medium did not affect the *λ*_max⁡_ of ABZ. The percentages of dissolved drug were statistically analyzed by a one-way analysis of variance. Differences were considered statistically significant at *P* < 0.05.

#### 2.2.5. *In Vivo* Assessment: Pharmacokinetic Study

(*1) Experimental Animals.* Six healthy (3–6-year-old) parasite-free female Retriever dogs, weighing 25–35 Kg, were used in this trial. Whole dogs were fed 12 h before the treatment and refed 12 h upon treatment. Experimental dogs were randomly allocated into two groups (*n* = 3) (group I: animals number 1, number 2, number 3; group II: animals number 4, number 5, number 6), which received two different treatments using a crossover design. Each experimental treatment was given to the six animals in two phases: phase I: animals in Group I received ABZ at 25 mg/kg as CTs (treatment A). Animals in Group II received 25 mg/kg of ABZ vehiculized in RDT (treatment B). After 21 days of a wash-out period, both treatments were reversed and repeated as phase II.

Blood samples were collected from the antebrachial vein using a 18 G catheter before administration (time 0) and at 0.25, 0.5, 1, 2, 4, 8, 12, 18, and 24 h after the oral administrations of the respective formulations, before being immediately transferred into heparinized tubes. Blood samples were centrifuged at 2000×g for 15min and the recovered plasma was stored at −20°C until analysis by HPLC.

*(2) Analysis of ABZ and Its Metabolites.* Sample cleanup: ABZ, ABZSO, and ABZSO_2_ were extracted using disposable C18 columns. Five microliters of Oxfendazole (OBZ) (5 *µ*g/mL) was added to 100 *µ*L of plasma in a glass test tube. Spiked samples were placed into a C18 column preconditioned with 0.5 mL of methanol followed by 0.5 mL water, in a vacuum system. Samples were washed (2 mL of water) and then eluted with 2 mL of HPLC-grade methanol. After elution, all samples were concentrated to dryness in a vacuum concentrator and then reconstituted with 150 *µ*L of the mobile phase.

*HPLC Analysis.* Experimental and spiked plasma samples (used for validation) were analysed by HPLC using UV detection. 

Chromatography was performed on a Shimadzu HPLC equipment (Shimadzu Corporation, Kyoto, Japan), provided with two LC-10AS solvent pumps, an automatic sample injector (SIL-10A) with a 50 *μ*L loop, an ultraviolet visible spectrophotometric detector (UV) (SPD-10A) read at 292 nm, a column oven (Eppendorf TC-45, Eppendorf, Madison, WI, USA) set at 30°C, and a CBM-10A integrator. Data and chromatograms were collected and analyzed using the Class LC10 software (SPD-10A, Shimadzu Corporation, Kyoto, Japan). The C18 reversed-phase column (5 *μ*m, 250 mm × 4.6 mm) was obtained from Kromasil (Kromasil, Sweden). Elution from the stationary phase was carried out at a flow rate of 1.2 mL/min using acetonitrile (40%) and a potassium phosphate buffer (25 mM, pH 5.3, 40%) as the mobile phase.

Fifty microliters of each sample was injected, and the analytes were eluted from the analytical column (5 *μ*m, 250 mm × 4.6 mm, C18 column) using a linear gradient method as reported by Sánchez et al. [21]. The compounds were identified by the retention times of standard references. Plasma calibration curves for each analyte were constructed by a least squares linear regression analysis, which gave a correlation coefficient (*r*) of between 0.9987 and 0.9995. The limits of quantification were 0.01 *μ*g/mL (ABZ and ABZSO) and 0.03 *μ*g/mL (ABZSO_2_).

*Pharmacokinetic Analysis of the Data.* The concentration versus time curves for the metabolites ABZSO and ABZSO_2_ in plasma for each individual animal after the different treatments were fitted using PK Solution 2.0 (Summit research services, Ashland, OH, USA). The following equation [22] was used to describe the biexponential concentration-time curves for ABZSO and ABZSO_2_ after the oral treatment:

(2) $$\begin{array}{c} C_{p}=Be^{-\lambda_{2}\cdot t}-Be^{-\lambda_{1}\cdot t}, \end{array}$$

where *C*_*p*_ is concentration in plasma at time *t* after administration (*µ*g/mL); *B* is concentration at time zero, extrapolated from the elimination phase (*µ*g/mL); *e* is base of the natural logarithm; *λ*_2_ is terminal slope (h^−1^); and *λ*_1_ is the slope obtained by feathering, which represents either the first-order absorption rate constant (*λ*_1_) or the first-order metabolite formation rate constant (*λ*_for_) (h^−1^). The elimination half-life (*t*_1/2_ *λ*_2_) and absorption (*t*_1/2_ *λ*_1_) or the metabolite formation half-lives (*t*_1/2_ *λ*_for_) were calculated as ln⁡2/*λ*_2_ and ln⁡2/*λ*_1_, respectively. 

The peak concentration (*C*_max⁡_) and time to peak concentration (*T*_max⁡_) were obtained from the plotted concentration-time curve of each analyte. The area under the concentration-time curve (AUC) and the area under the first moment curve (AUMC) were calculated by the linear trapezoidal rule [23]:

(3) $$\begin{array}{c} \text{AUM}{\text{C}}_{\left(\text{0}-\infty \right)}=\sum_{i=0}^{n-1}\frac{t_{i+1}-t_{i}}{2}\left(c_{i}t_{i}+c_{i+1}t_{i+1}\right)\left(\frac{C_{\text{last}}*t_{\text{last}}}{\lambda_{z}}+\frac{C_{\text{last}}}{\lambda_{z}^{2}}\right). \end{array}$$

*Statistical Analysis of the Data.* The nonparametric Mann-Whitney test was used for the multiple statistical comparisons of the PK data obtained from the different groups. A value of *P* < 0.05 was considered to be statistically significant.

## 3. Results and Discussion 

Powder blends easily compacted by means of direct compression should possess good flow and compression properties. The rheological properties of powder blends were assayed by evaluation of density, angle of repose, and compressibility (Hausner ratio). The Hausner ratio and Carr's index refer to the packing characteristics of the materials and therefore can be used as indicators of powder flowability. For both RDT and CT formulations, the results (Table 2) showed that the powders possessed suitable rheological properties and compressibility. The hardness, disintegration, and friability of the compacts were also evaluated for each formulation (Table 3) because tablet disintegration as well as drug dissolution is relevant, with both being closely related to the expected biopharmaceutical performance of the formulation. As expected, the disintegration time was shorter in the case of RDTs in comparison to the CTs. This may have been attributed to the incorporation of Ludiflash into the formulae, which was then able to produce water uptake, swelling, and quick rupture of the compact. For both formulations and for all the compaction forces, the friability was less than 1%.

Regarding the compression behaviour, the influence of compaction forces (CF) on the hardness and the disintegration time of the compact was analysed. When the CF was raised, an increase of the hardness in the tablets was observed, as expected. This was remarkable when CTs were considered, due to the presence of Avicel, which is a very compressible material.

**Figure 1:**
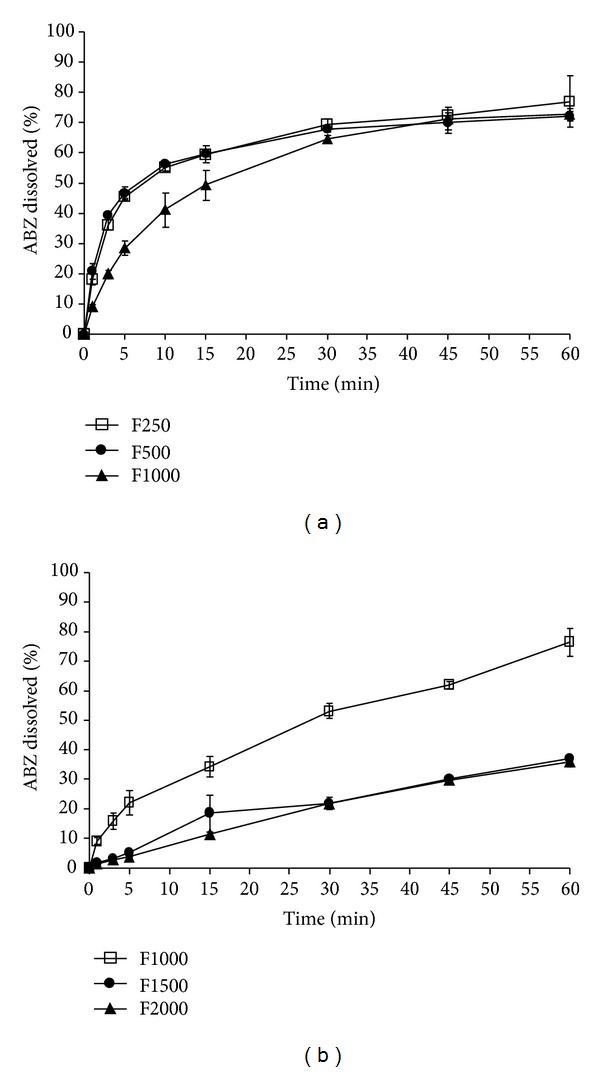
Dissolution profile: (a) rapidly disintegrating tablets (RDTs) and (b) conventional tablets (CTs) at different compaction forces: F250: compaction forces a 250 mPa; F500: compaction forces a 500 mPa; F1000: compaction forces a 1000 mPa; F1500: compaction forces a 1500 mPa; F2000: compaction forces a 2000 mPa.

The *in vitro* ABZ dissolution profiles are shown in Figures 1(a) and 1(b). In particular the RDTs dissolution rate was not affected by increasing the CF, when tablets were compacted at 250 and 500 mPa. However, at 1000 mPa a slower dissolution rate was visualized, which was a consequence of the observed enhancement of the disintegration time. The CTs compacted at 1000 MPa showed the fastest dissolution rate, as observed in Figure 1(b). 

From these results, and based on the lower hardness/disintegration and faster dissolution rate, CTs compacted at 1000 mPa and RDTs compacted at 500 mPa were selected as the model formulations for further *in vivo* bioavailability studies.

Differences in the pharmacokinetic profiles of benzimidazoles (BZD) anthelmintics have been reported, depending on the animal species. Anatomic features influence the passage of digesta and the bioavailability, so the pharmacokinetics of anthelmintics may be affected by different gut transit features [24]. ABZ parent drug was not detected in plasma, being the detection of the metabolite ABZSO (also named ricobendazole) as the main molecule with antiparasitic activity in bloodstream. In dogs, the plasma concentrations and the mean residence times of ABZ and their sulfoxide and sulphone metabolites are lower and shorter, respectively, in comparison to in ruminant species on human [25]. This may be explained by the fact that there exists a relatively short transit time in the gastrointestinal tract of dogs compared to other animal species.

According to various reports, the absorption of BZD anthelmintic drugs in cats, dogs, and humans is substantially limited by their low dissolution rates in gastric fluids [26, 27]. Consequently, these compounds must be administrated at high doses or as multiple doses in order to provide sustained concentrations at the parasite site. It is well known that the incorporation of low-solubility drugs into solid dispersions (SDs) is an effective alternative for increasing the *in vitro* dissolution rate. In this paper, we improved this property in ABZ by obtaining a solid dispersion compounded by ABZ : P 188 (1 : 1) [8], with an increase being observed in the dissolution assays when solid dispersions were poured as powder onto the dissolution media surface. However, when SDs were put into capsules, the corresponding dissolution test showed that the capsules were not able to improve the dissolution rate [9]. 

As a consequence of the thermal gelation properties of P 188, the partially compacted powder absorbed water produced swelling and gel formation and an increase in gel viscosity due to the rise of temperature (37°C). However, this behavior was practically negligible when SDs were manipulated as a bulk powder in similar conditions. In a previous work, carried out on mice, we observed a significant increase in the bioavailability when solid dispersions were administered orally as a multiparticulate system (thus avoiding the effects of compaction) compared to ABZ suspension [28].

**Figure 2:**
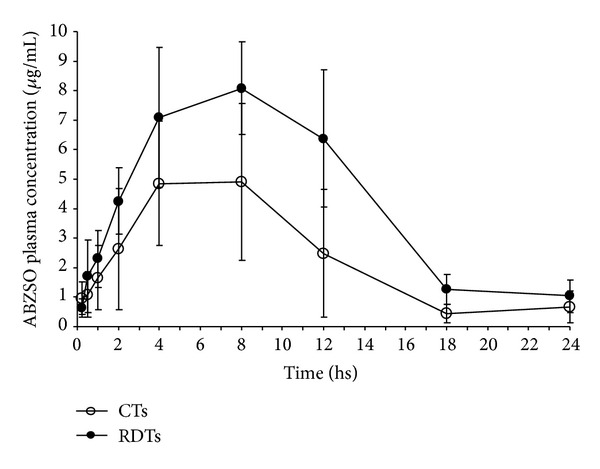
Comparative (mean ± SD) plasma profiles for albendazole sulfoxide (ABZSO) after the administration of two different oral-based albendazole (ABZ) formulations: rapidly disintegrating tablets (RDTs) and conventional tablets (CTs).

**Figure 3:**
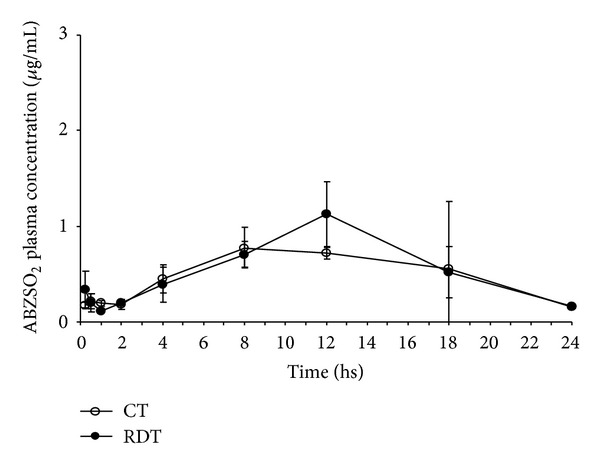
Comparative (mean ± SD) plasma profiles for albendazole sulphone (ABZSO_2_) after the administration of two different oral-based albendazole (ABZ) formulations: rapidly disintegrating tablets (RDTs) and conventional tablets (CTs).

From these results and considering that the formulation of a tablet may imply a compaction process that decisively affects the dissolution rate, we investigated the incorporation of ABZ solid dispersion in the RDTs (treatment B), as a viable alternative for avoiding or minimizing adverse effects. Therefore, the formulation with ABZ incorporated in CTs (treatment A) was studied for its effect on the bioavailability in dogs (Figures 2 and 3, Table 4). 

In our case, the results obtained *in vitro* were reflected in the *in vivo* studies. 

As reported in other studies, after both treatments the ABZ parent drug was not detected in the plasma, but ABZSO was detected in plasma at 18 h and 24 h after treatment (treatments A and B, respectively). This ABZSO plasma disposition (AUC) was significantly greater (1.6-fold more at *P* < 0.05) after treatment B compared to treatment A. This same trend was observed for parameter *C*_max⁡_ after treatment B, where the concentration obtained was 1.5-fold higher compared with treatment A (*P* < 0.05). A nonsignificant delay in *T*_max._ was present after treatment B (7.2 h) compared with treatment A (6 h) (see Table 4 and Figure 2).

Modifications of the pharmacokinetic parameters were reflected in an improvement of the dissolution rate as a consequence of the rapid disintegration of the RDTs, which produced a remarkable increase in kinetic absorption as well as in the drug amount available in the plasma. The RDTs were able to disintegrate in only a few seconds after administration, allowing the resulting fine solid dispersion to reach the stomach where the rapid dissolution of solids may occur. In contrast, each solid particle compacted in CTs was very difficult to be accessed by the solvent, and a process of swelling and gelation began. Thus, the gel layer formed prevented the particle from being wetted and resulted in disintegration and drug dissolution. This delay in the ABZ dissolution process was especially deleterious for ABZ bioavailability, since it has to dissolve rapidly along the short length of the dog's gastrointestinal tract.

## 4. Conclusions

The bioavailability of ABZ from SD compacted in tablets was extremely conditioned by the initial dissolution rate. A tablet design based on fast disintegrating excipients was shown to be more successful in increasing the absorption process compared with traditional tablets. Therefore, rapid disintegration of tablets seems to be a key factor for efficiency of SD with regard to improvement of the ABZ bioavailability. 

The *in vivo* assays performed on dogs showed a marked increase in drug plasma exposure when ABZ was given in SD incorporated into RDTs compared with CTs. This behavior allowed us to deduce that a positive and direct correlation existed between the *in vitro* dissolution rate and the expected pharmacokinetic of ABZ. Although SD was demonstrated to be an efficient strategy for improving the dissolution rate, a correct formulation of the tablet was necessary in order to guarantee that the disintegration process was not a limiting step in the ABZ absorption. This formulation may have a beneficial impact by shortening the antiparasite therapy time needed in monogastrics. 

## Figures and Tables

**Table 1:** Components of the rapidly disintegrating tablets (RDTs) and conventional tablets (CTs).

Ingredients (mg)	Rapidly disintegrating tablets	Conventional tablets
Albendazole	—	200
Solid dispersions	400	—
Lactose/Avicel	—	25/75
Ludiflash	732	—
Sodium crosscarmellose	60	67.2
Colloidal silicon dioxide	—	8
Magnesium stearate	8	16

**Table 2:** Physical-mechanical characterization of the formulations: Bulk density (BD), Tap density (TD), Carr's index, Hausner ratio, and angle of repose.

	Angle of repose (°)	Carr's index (%)	Hausner Ratio (%)	TD (m/v)	BD (m/v)
Conventional tablets	39 ± 2	16.6	1.2	0.6 ± 0.02	0.5 ± 0.1
Rapidly disintegrating tablets	38.9 ± 1.65	22.22	1.28	0.63 ± 0.02	0.49 ± 0.01

**Table 3:** Values of hardness and disintegration time for both formulations at different compaction forces.

	Compaction force (mPa)	Hardness (kg/cm^2^)	Disintegration time (min.)
Conventional tablets	1000	4 ± 1	5.5
	1500	11 ± 1	12
	2000	18 ± 3	15

Rapidly disintegrating tablets	250	3 ± 0.5	2.5
	500	4 ± 0.5	2.5
	1000	8 ± 2	2.7

**Table 4:** Comparative plasma disposition kinetic variables for albendazole sulfoxide (ABZSO) and albendazole sulphone (ABZSO_2_) after the oral administration of three different formulations.

Pk parameters	Group I (ABZ control) conventional tablets		Group II Rapidly disintegrating tablets	
	ABZSO	ABZSO_2_	ABZSO	ABZSO_2_
*T*_1/2 abs._ (h)	2.14 ± 1.28	2.46 ± 1.81	2.38 ± 0.42	4.93 ± 1.81
*T*_max⁡_ (h)	6.00 ± 2.31	7.13 ± 4.80	7.20 ± 1.79	12 ± 0.00
*T*_1/2 elim._ (h)	4.67 ± 1.68	32.13 ± 39.63	4.73 ± 1.19	10.86 ± 7.47
*C*_max⁡_ (*µ*g/mL)	5.33 ± 2.83	0.67 ± 0.27	8.18* ± 1.47	1.13 ± 0.34
AUC_0–*∞*_ (*µ*g·h/mL)	52.20 ± 12.95	33.32 ± 46.20	113.11* ± 22.85	22.32 ± 10.33
AUMC_0–*∞*_ (*µ*g·h^2^/mL)	718.50 ± 274.17	222.56 ± 149.04	1180.30 ± 306.44	382.18 ± 344.47
MRT (hr)	12.11 ± 3.99	49.76 ± 56.09	10.48 ± 1.78	21.71 ± 10.90
PDP (h)	0.25–24.0	0.25–24.00	0.25–24.0	0.25–24.0

*T*_1/2  abs._: metabolite formation half-life, *C*_max⁡_: peak concentration, *T*_max⁡_: time at *C*_max⁡_, *T*_1/2  elim._: elimination half life, AUC_0–*∞*_: area under the concentration versus time curve extrapolated to infinity, AUMC_0–*∞*_: area under the moment of the concentration versus time curve extrapolated to infinity, MRT: Mean residence time, and PDP: plasma detection period. (*) values are statistically different to group I at *P* < 0.05.

## Appendix

Animal procedures and management protocols were approved by the Ethics Committee according to the Animal Welfare Policy (act 087/02) of the Faculty of Veterinary Medicine, Universidad Nacional del Centro de la Provincia de Buenos Aires (UNCPBA), Tandil, Argentina (http://www.vet.unicen.edu.ar/).
